# Supplementary material for: Identification of Proteins Differentially Expressed by Adipose-derived Mesenchymal Stem Cells Isolated from Immunodeficient Mice
Source: Int J Mol Sci. 2019 May 30;20(11):2672. doi: 10.3390/ijms20112672 (PMC6600271; doi:10.3390/ijms20112672)
Supplement: Supplementary file 1 [file ijms-20-02672-s001.zip › ijms-475474 supplementary/ijms-475474 supplementary figures.pdf]

A

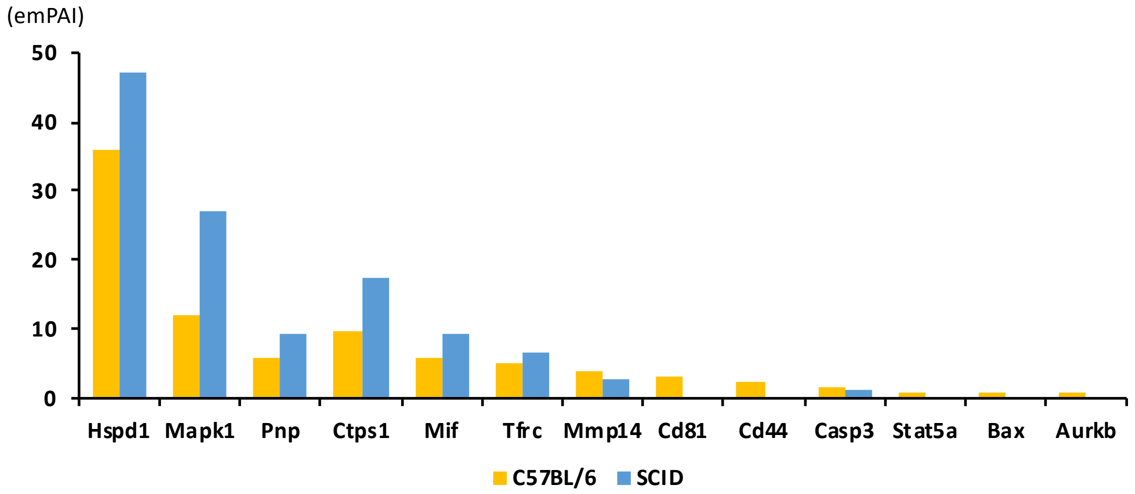

B

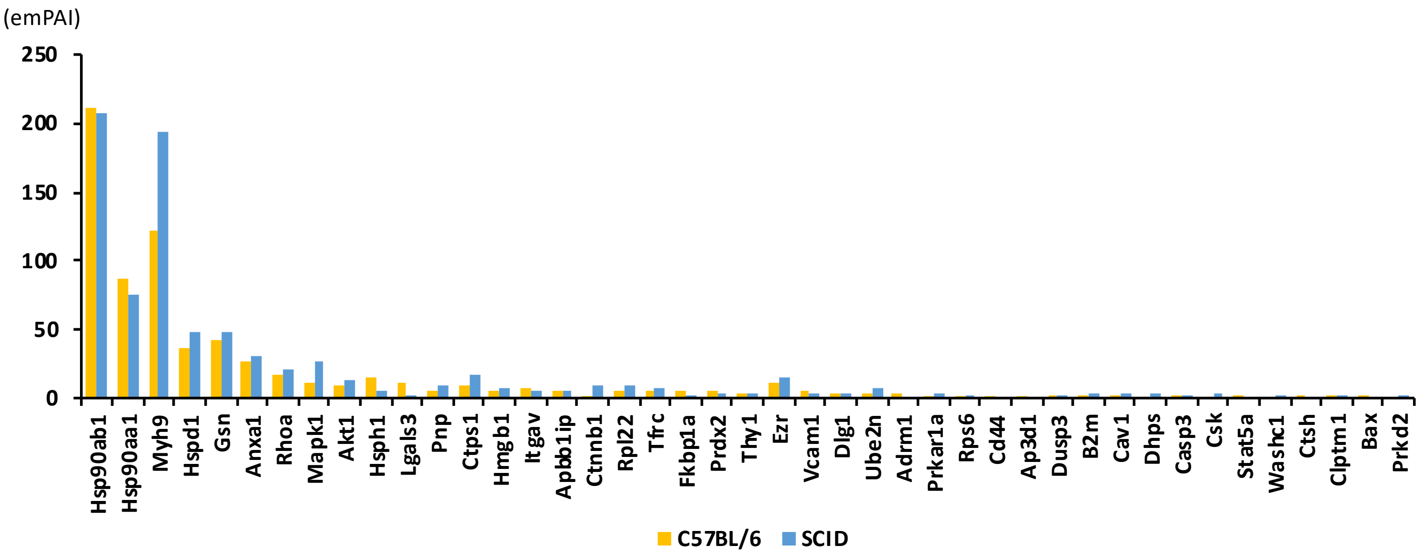

Supplementary Figure 1. Evaluation of the clusters of B and T cell-related proteins according to the normalized emPAI values of each housekeeping gene (HKG) expressed by C57BL/6 and SCID mAdMSCs.

A

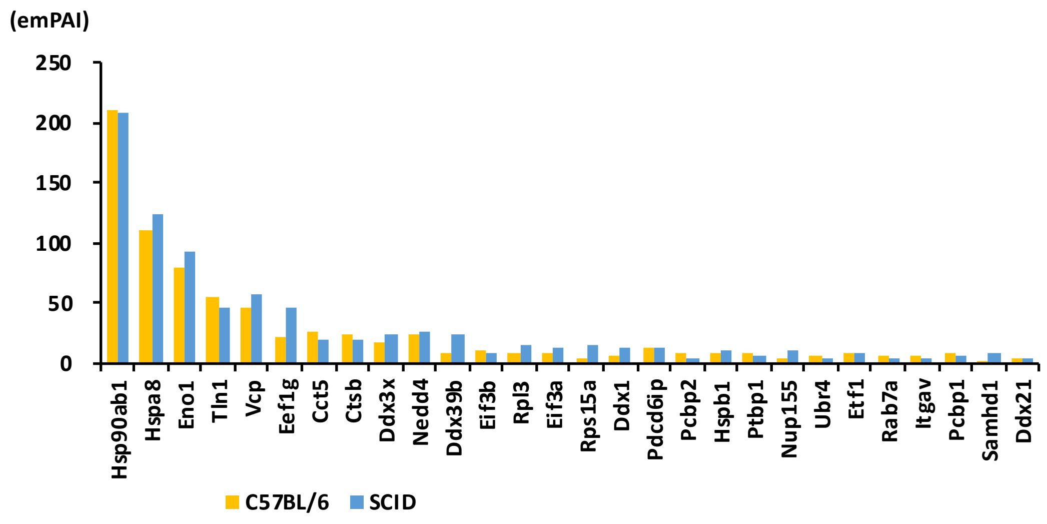

B

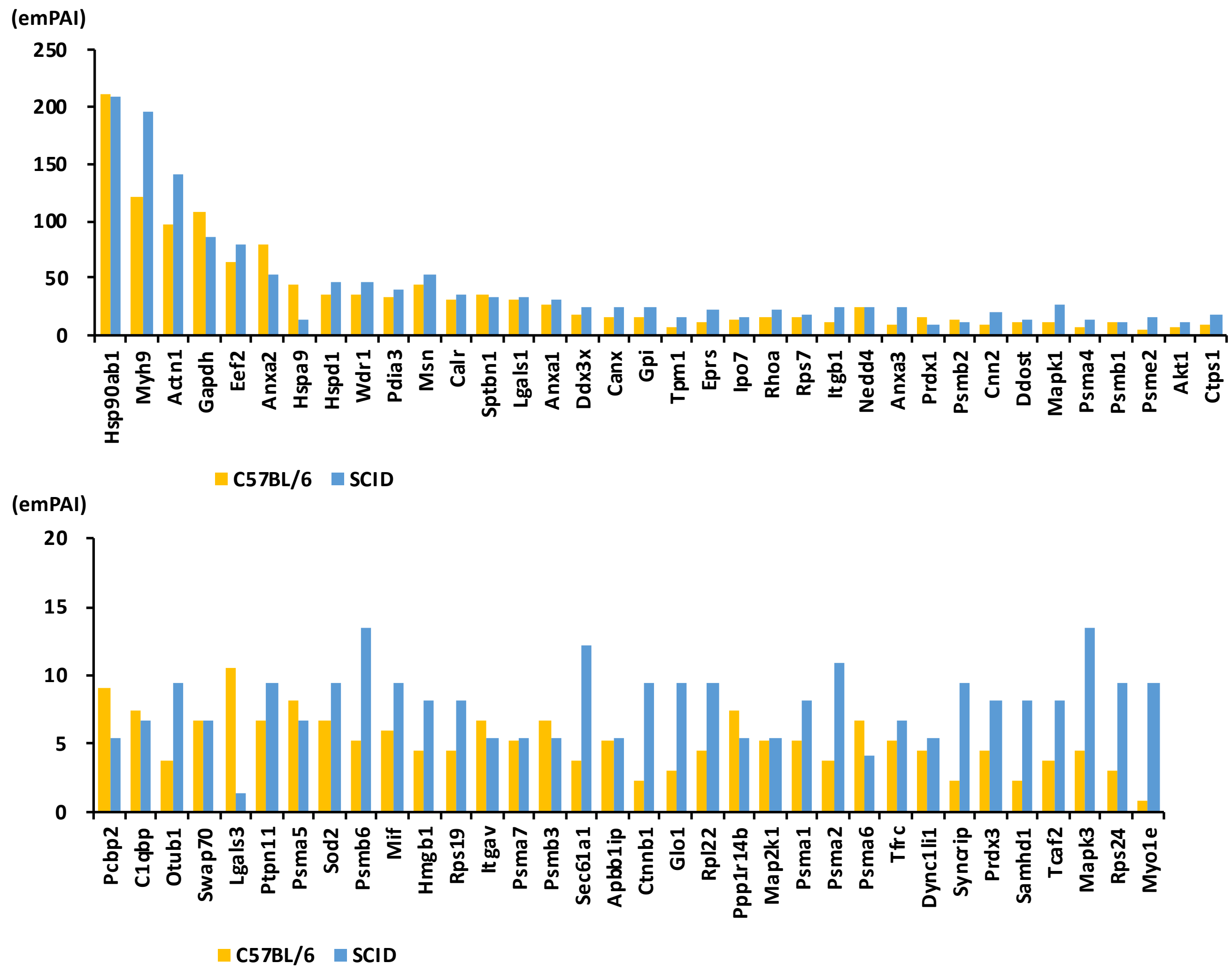

Supplementary Figure 2. Evaluation of the cluster of viral process-related proteins and immune system process-related proteins.

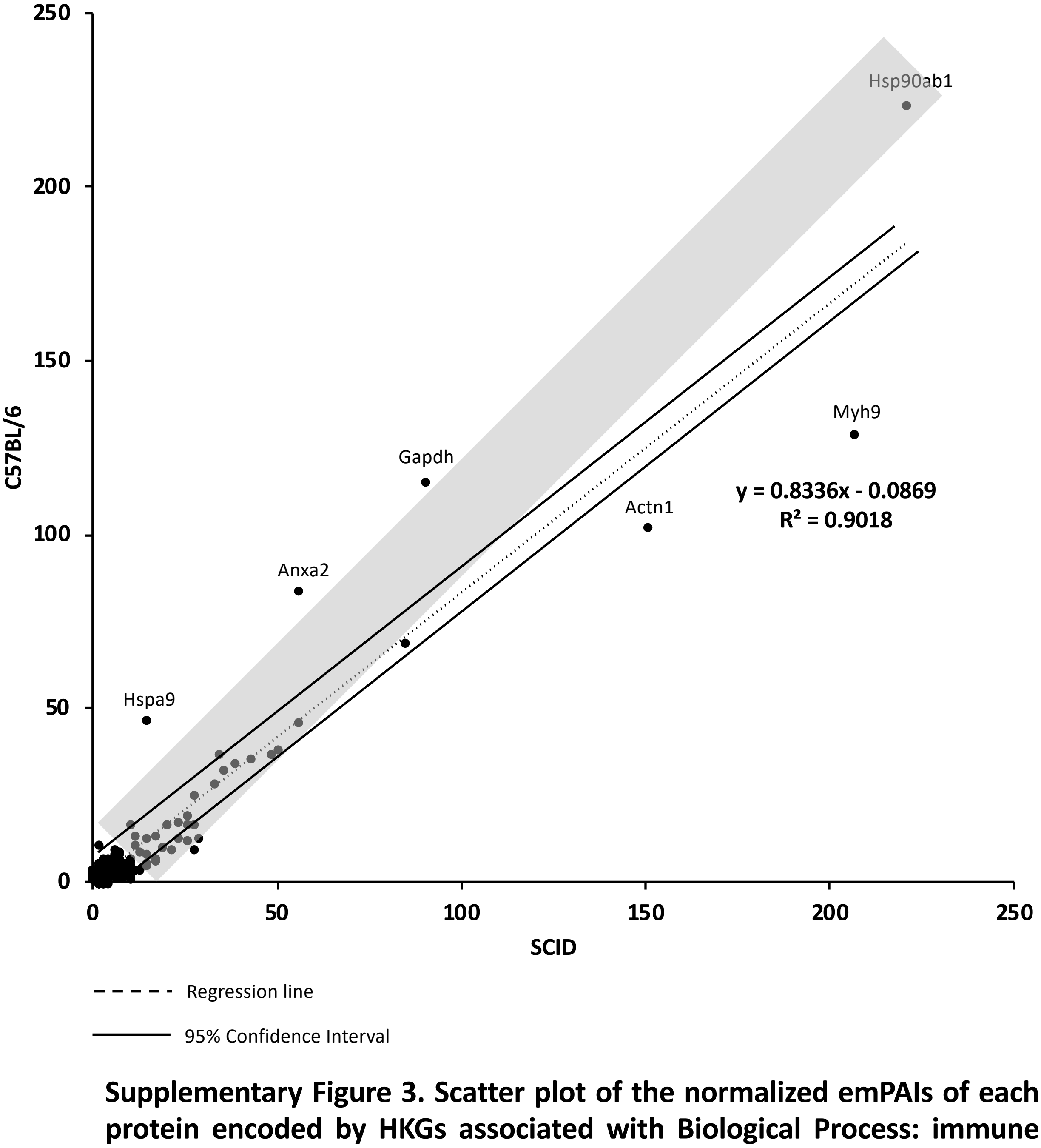

Supplementary Figure 3. Scatter plot of the normalized emPAIs of each protein encoded by HKGs associated with Biological Process: immune system process).

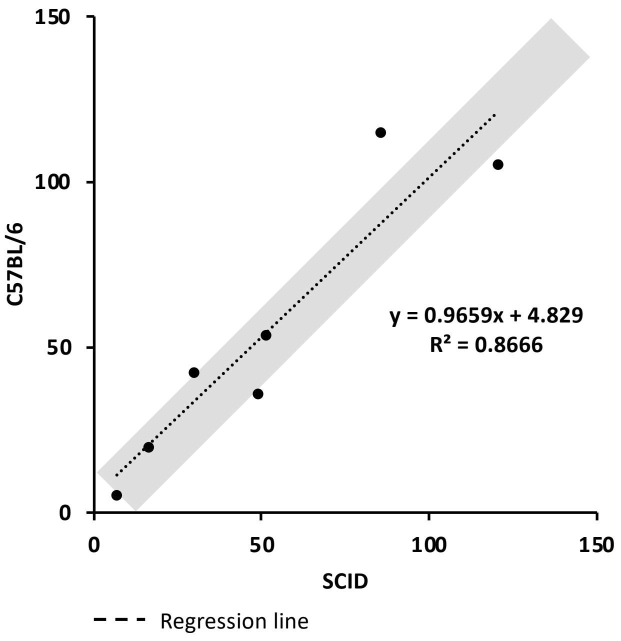

Supplementary Figure 4. Scatter plot of the values of proteins encoded by HKGs.
